# Inhibition of Hippo Signaling Improves Skin Lesions in a Rosacea-Like Mouse Model

**DOI:** 10.3390/ijms22020931

**Published:** 2021-01-19

**Authors:** Jihyun Lee, Yujin Jung, Seo won Jeong, Ga Hee Jeong, Gue Tae Moon, Miri Kim

**Affiliations:** 1 Department of Dermatology, Seoul St. Mary’s Hospital, College of Medicine, The Catholic University of Korea, #222 Banpo-daero, Seocho-gu, Seoul 06591, Korea; yiji1@hanmail.net; 2 Department of Biomedicine & Health Sciences, The Catholic University of Korea, #222 Banpo-daero, Seocho-gu, Seoul 06591, Korea; worldgh27@naver.com (G.H.J.); therlone@naver.com (G.T.M.); 3 Department of Dermatology, Yeouido St. Mary’s Hospital, College of Medicine, The Catholic University of Korea, #10, 63-ro, Yeongdeungpo-gu, Seoul 07345, Korea; yjung9025@gmail.com (Y.J.); cmcos@hanmail.net (S.w.J.)

**Keywords:** rosacea, hippo signaling, yes-associated protein (YAP), transcription activator with a PDZ-binding motif (TAZ), angiogenesis

## Abstract

The Hippo signaling pathway plays a key role in regulating organ size and tissue homeostasis. Hippo and two of its main effectors, yes-associated protein (YAP) and WWTR1 (WW domain-containing transcription regulator 1, commonly listed as TAZ), play critical roles in angiogenesis. This study investigated the role of the Hippo signaling pathway in the pathogenesis of rosacea. We performed immunohistochemical analyses to compare the expression levels of YAP and TAZ between rosacea skin and normal skin in humans. Furthermore, we used a rosacea-like BALB/c mouse model induced by LL-37 injections to determine the roles of YAP and TAZ in rosacea in vivo. We found that the expression levels of YAP and TAZ were upregulated in patients with rosacea. In the rosacea-like mouse model, we observed that the clinical features of rosacea, including telangiectasia and erythema, improved after the injection of a YAP/TAZ inhibitor. Additionally, treatment with a YAP/TAZ inhibitor reduced the expression levels of YAP and TAZ and diminished vascular endothelial growth factor (VEGF) immunoreactivity in the rosacea-like mouse model. Our findings suggest that YAP/TAZ inhibitors can attenuate angiogenesis associated with the pathogenesis of rosacea and that both YAP and TAZ are potential therapeutic targets for patients with rosacea.

## 1. Introduction

Rosacea, a chronic inflammatory cutaneous condition characterized by facial erythema and telangiectasia, affects approximately 5–10% of the population [1]. Although the pathophysiology of rosacea remains unclear, multiple factors, including an altered innate immune system, dermal matrix degeneration, antimicrobial peptide dysfunction, and increased neoangiogenesis, have been implicated in its pathogenesis [2,3,4]. Previous studies have shown increased numbers of blood vessels, enlargement of blood vessels, and elevation of vascular endothelial growth factor (VEGF) in the skin lesions of patients with rosacea [5,6,7]. Cuevas et al. [8] reported improvements in erythema and telangiectasia following the use of topical dobesilate, an inhibitor of angiogenic growth factor that is effective for the treatment of erythematotelangiectatic rosacea [9].

The Hippo signaling pathway is a principal regulator of tissue development and homeostasis [10], and its critical roles in vascular development have been recently noted [11,12]. The Hippo signaling effectors yes-associated protein (YAP) and TAZ (transcriptional coactivator with PDZ-binding motif)—also known as WW domain-containing transcription regulator 1 (WWTR1) or (WWTR1/TAZ)—have been implicated in the proliferation, sprouting, and maturation of blood vessels [11,13,14]. Because the blockade of YAP inhibits the expression of VEGF, the roles of these effector molecules were investigated to determine the underlying mechanisms of vascular abnormality and growth of new vessels observed in diabetic retinopathy [9]. Therefore, YAP/TAZ could be therapeutic targets for the treatment of angiogenesis-related diseases. In particular, the inhibition of YAP/TAZ may exert a therapeutic effect on rosacea through anti-angiogenesis and blockade of Hippo signaling.

However, it is unclear whether Hippo signaling is involved in the pathogenesis of rosacea. This study explored the expression of YAP and TAZ, downstream effectors of Hippo signaling, in human and mouse samples; identified whether the inhibition of YAP/TAZ in mice improved the rosacea-like phenotype; and evaluated the histological and molecular changes in a rosacea mouse model after the application of YAP/TAZ inhibitors.

## 2. Results

### 2.1. Elevated YAP and TAZ Expression in Skin Samples from Patients with Rosacea

**Figure 1:**
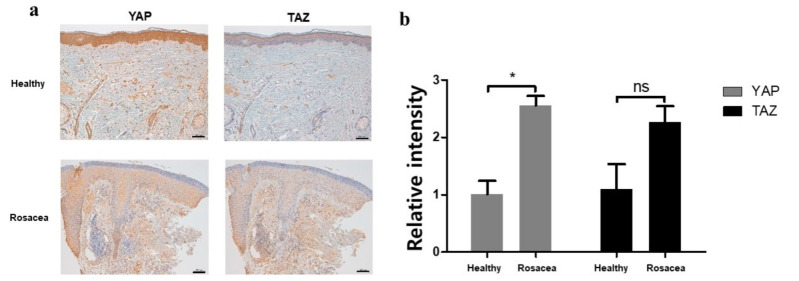
(**a**) Skin samples from patients with rosacea showed high levels of yes-associated protein (YAP) and transcription activator with a PDZ-binding motif (TAZ), compared with samples from healthy volunteers. Original magnification, ×200; scale bar = 100 μm. (**b**) Immunostaining intensities were calculated with ImageJ software. * means data was significant (*p* < 0.05), and ns means nonsignificant.

To determine whether the expression levels of YAP and TAZ differed in patients with rosacea, skin samples were obtained from lesions of patients with rosacea and from similar locations in healthy volunteers. The samples were stained using antibodies directed against YAP and TAZ, respectively. Overall, tissue samples from patients with rosacea demonstrated a diffusely larger and darker brown stain uptake compared with samples from healthy volunteers, as shown in Figure 1a. To quantify the differences, immunostaining intensities were calculated with ImageJ software. The findings demonstrate significantly enhanced dermal staining for both YAP and TAZ in tissues from patients with rosacea, compared with healthy volunteers. Although the increase in TAZ expression was not statistically significant, TAZ expression intensity was higher in the rosacea group than healthy volunteers (Figure 1b).

### 2.2. Alleviation of the Clinical Symptoms of Rosacea in BALB/c Mice with Verteporfin (VP)

**Figure 2:**
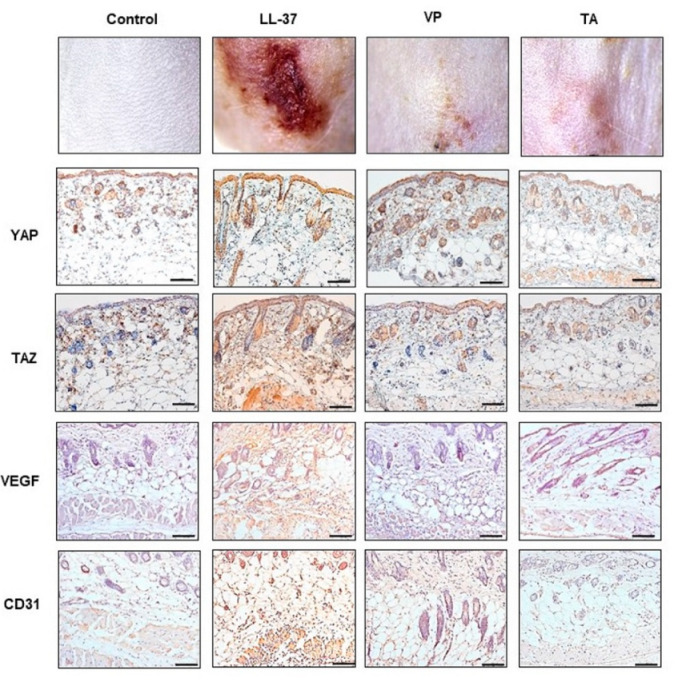
The LL-37-induced rosacea-like mouse model. Representative photomicrographs of YAP- and TAZ-, hematoxylin and eosin (H&E)-, and vascular endothelial growth factor (VEGF)-stained skin sections. Original magnification, X200; scale bar = 100 μm.

VP interferes specifically with the reciprocal interaction between YAP and the transcription factor TEAD, thereby inhibiting TAP function [15]. A common antimicrobial peptide in rosacea, LL-37, enhances endothelial cell angiogenesis and proliferation and promotes inflammation [4]. To determine whether VP could improve the clinical features of rosacea by repressing interactions involved in the Hippo/YAP pathway, rats were injected with VP. As shown in Figure 2, treatment with VP markedly alleviated the clinical signs of rosacea, including erythema, vasodilation, and telangiectasia, compared with the LL-37-injected rosacea group. The clinical range of erythema was even smaller than in the group treated with triamcinolone (TA), which is commonly used to reduce inflammation.

### 2.3. Suppression of Angiogenesis by VP

Because VP improved telangiectasia and erythema, hematoxylin and eosin (H&E)-stained skin sections were examined to determine whether inhibition of YAP/TAZ could induce histological changes. Antibodies directed against YAP, TAZ, VEGF, and CD31 were used to stain tissues from control, LL-37-, VP-, and TA-treated mice. Consistent with our findings of greater YAP and TAZ expression in patients with rosacea, the overall YAP and TAZ expression patterns were more diffuse in LL-37-induced rosacea-like mice. Compared with the control group, H&E staining revealed that injection with LL-37 induced more vessel proliferation, vasodilation, and dense inflammatory cell infiltration (Figure 2), similar to the histological changes in patients with rosacea. In contrast, administration of VP to the LL-37-injected group resulted in reduced inflammatory cell infiltration and vessel density in the dermis, similar to treatment with TA (Figure 2). In addition, dermal VEGF immunoreactivity, which is associated with angiogenesis, was measured by H&E staining. As indicated by the intensity of brown coloration (Figure 2), VEGF immunoreactivity was significantly denser and more diffuse in the LL-37-induced rosacea-like group compared with the control group. Conversely, treatment with VP reduced the VEGF immunohistochemical staining intensity in LL-37-induced rosacea-like mice (Figure 2).

### 2.4. Significant Reduction in VEGF mRNA Expression after VP Application

**Figure 3:**
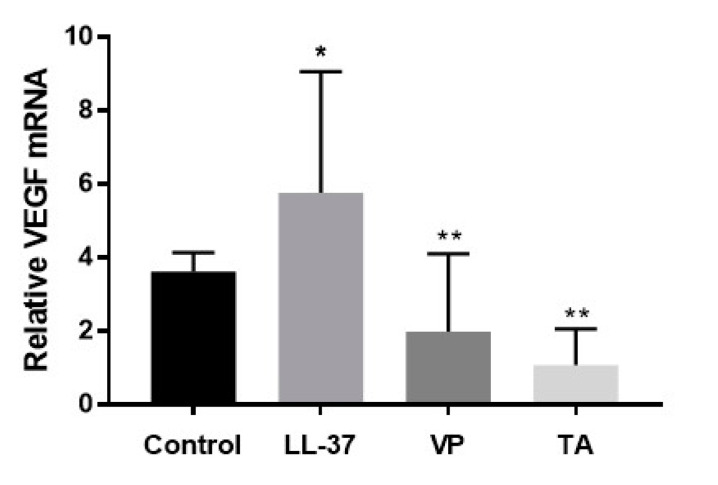
Quantitative polymerase chain reaction (qPCR) analyses of VEGF mRNA expression. Data represent the means and SEMs of three independent experiments (*n* = 3 for the control group and *n* = 5–6 for the other groups). * *p* < 0.05 (control group vs. LL-37 group), ** *p* < 0.01 (LL-37 group vs. verteporfin (VP) and triamcinolone (TA) groups) (Mann–Whitney *U* test).

To further confirm whether VP-mediated inhibition of YAP/TAZ can affect the molecular mechanism underlying angiogenesis, we analyzed the mRNA expression levels of vascular markers such as VEGF using quantitative polymerase chain reaction (qPCR). The mRNA level of VEGF in the LL-37-induced rosacea-like group was significantly greater than in the control group, implying that VEGF-associated angiogenesis was enhanced by rosacea. Similar to the TA group, treatment with VP significantly reduced VEGF mRNA expression, compared with expression in the rosacea group. VP noticeably inhibited the expression levels of YAP/TAZ target genes and VEGF-associated molecular reactions, suggesting that the vasoconstrictor activity of VP in human skin vessels is able to modulate erythema and telangiectasia in rosacea (Figure 3).

## 3. Discussion

Rosacea is a chronic inflammatory skin disease characterized by centro-facial erythema, telangiectasia, papules, and pustules. The typical histological findings of rosacea include an elevated number of superficial blood vessels with enlarged and dilated lumens, inflammatory cell infiltration, and solar elastosis [15]. VEGF, fibroblast growth factor-2, and matrix metalloproteinase-1 contribute to disease onset by inducing remodeling of the vasculature and dermal matrix [16]. YAP and TAZ, members of the Hippo signaling pathway, are strongly expressed in vascular proliferative and inflammatory skin diseases [17]. They are also involved in the proliferation, sprouting, and maturation of blood vessels [14,18], crucial components of rosacea pathogenesis.

Here, we used an LL-37-induced mouse model of rosacea to determine whether YAP/TAZ blockade could influence the severity of rosacea-like skin inflammation and angiogenesis. The histological and molecular results demonstrate that YAP/TAZ inhibitors can modulate rosacea. The effects of inhibition were demonstrated in terms of fewer blood vessels and reduced VEGF mRNA expression, both important factors in rosacea pathogenesis.

Notably, we found that YAP/TAZ expression levels were significantly greater in patients with rosacea than in healthy volunteers, indicating that YAP/TAZ play roles in disease onset and progression. Elevated YAP/TAZ expression levels in rosacea tissues suggest that these proteins induce vascular development during rosacea pathogenesis. These findings are consistent with the results of a previous study that showed that YAP/TAZ play critical roles in angiogenesis and vascular barrier maturation in terms of modulating actin cytoskeleton remodeling, coordinating vascular endothelial cells, and regulating the expression of extracellular matrix proteins [19].

To explore the in vivo therapeutic effects of YAP/TAZ inhibitors, we induced a rosacea-like clinical phenotype using LL-37, as described previously [4]. As in previous reports [2,4], the LL-37-injected skin lesions exhibited rosacea-like cutaneous phenotypes such as erythema and telangiectasia. VP, a photosensitizer initially designed for photodynamic therapy, is an inhibitor of YAP/TAZ interactions as well as their transcriptional activities [19]. These clinical features were notably improved in the VP-treated group.

The mechanism underlying the inhibitory effect of VP on angiogenesis has not been studied at the molecular level. To further investigate the mechanism underlying inhibition of vascular proliferation through VP, we performed immunostaining and qPCR analyses of VEGF, a major driver of blood vessel formation through endothelial cell proliferation and migration, which manifests as telangiectasia in affected patients [20]. Furthermore, elevated VEGF expression has been reported in the skin lesions of patients with rosacea [2,5]. In the present study, markedly upregulated VEGF expression was detected in the LL-37 group. Treatment with VP resulted in reduced VEGF expression, similar to treatment with TA. Additionally, the reduction in CD31+ reactivity in the VP group, compared with the LL-37 group, supports the hypothesis of VP-mediated inhibition. In particular, CD31 is reportedly associated with microvessels that contain immature endothelium, which is related to neo-angiogenesis. These findings imply critical roles for YAP/TAZ during angiogenesis in vivo [12] because the inhibition of YAP/TAZ by VP treatment effectively reduced VEGF-mediated vascular formation in rosacea-like mouse endothelial cells.

Furthermore, the Hippo signaling pathway and its downstream effectors, YAP/TAZ, reportedly contribute to angiogenesis and remodeling of the extracellular matrix through extracellular-signal-regulated kinase, which modulates VEGF [14,18,21]. Wang et al. [22] showed that VEGF controls the angiogenesis-related activation of YAP/TAZ through the actin cytoskeleton, while defects in YAP/TAZ caused a disrupted cellular distribution of VEGF receptors through plasma membrane trafficking defects. Consistent with previous reports, we found that VEGF expression was elevated in rosacea-like mice, implying that it serves as a downstream target of the Hippo pathway involved in the pathogenesis of rosacea. In addition, the clinical improvement in rosacea-like skin lesions mediated by the inhibition of YAP/TAZ indicates critical roles in the development of rosacea in association with VEGF-related angiogenesis.

In conclusion, treatment with VP, a YAP/TAZ inhibitor, reduced both clinical erythema and histologic vascular proliferation in a rosacea-like BALB/c mouse model. Our results indicate that the blockade of YAP/TAZ contributes to anti-angiogenic responses in rosacea and VEGF-related angiogenesis. We suggest that YAP/TAZ can serve as a therapeutic target for rosacea and other inflammatory skin diseases associated with angiogenesis. Additional studies are required to explore the precise mechanisms by which VP regulates angiogenesis and to determine whether VP is effective against rosacea in human patients.

## 4. Materials and Methods

### 4.1. Patient Samples

To examine YAP and TAZ expression patterns in patients with rosacea, skin biopsies were collected from inflammatory papules located on the cheeks of patients (*n* = 15) and from skin lesions of age-matched normal healthy volunteers (*n* = 9). Individuals were excluded from the study if they had been treated with drugs (e.g., antibiotics or isotretinoin) or had received laser treatment within 3 months prior to the baseline visit. This study was approved by the Ethical Committee of the Catholic University of Korea (approval code: KC18SESI0486; approval date: 08/16/2018), and all participants provided written informed consent.

### 4.2. Rosacea-Like Mouse Model

To explore the in vivo therapeutic efficacy of YAP/TAZ inhibitors, we induced a rosacea-like clinical phenotype using LL-37 peptides, as described previously [4]. Seven-week-old female BALB/c mice were purchased from DooYeol Biotech (Seoul, Korea) and acclimatized for 1 week before initiation of the experiment. All animals were maintained under specific pathogen-free conditions, and the study protocol was carried out in accordance with the guidelines of the Animal Care Committee of the Catholic University of Korea. The approval code of the ethics committee for the animal experiment was YEO20182701T. The mice were divided into four groups: control, LL-37 (i.e., rosacea-like), TA (positive control), and VP. The hair on the backs of the BALB/c mice was shaved using an electric razor. Twenty-four hours later, 40 μL of LL-37 in nanopure water (320 μM; InvivoGen, San Diego, CA, USA) was injected intradermally into the shaved area using a 0.5 mL insulin syringe (31 gauge), resulting in the formation of a dermal bleb. Control mice were injected intradermally with 40 μL of nanopure water alone. Immediately after intradermal injection, the LL-37 (LL-37 + phosphate-buffered saline), TA (LL-37 + TA), and VP groups (LL-37 + VP) were injected intraperitoneally with 200 μL of phosphate-buffered saline, TA (10 mg/kg in phosphate-buffered saline), and VP (Sigma-Aldrich, St. Louis, MO, USA; 100 mg/kg, dissolved in dimethyl sulfoxide), respectively. These procedures were repeated twice daily for 48 h, and the dorsal skin of each group was photographed after completion of the intraperitoneal injections. The severity of rosacea-like skin lesions was evaluated based on the redness score and involved area. The redness area was assessed by stereomicroscope measurements (Leica M50; Leica, Wetzlar, Germany). After treatment with or without VP, the BALB/c mice were anesthetized and sacrificed. Tissue samples of dorsal skin were then excised and specimens were divided for histological staining and gene expression analyses. Samples for tissue staining were fixed in 10% formalin and embedded in paraffin. The paraffin-embedded skin samples were sliced and then stained with H&E.

### 4.3. Immunohistochemical Analysis

Formalin-fixed, paraffin-embedded tissue sections (5 μm) were used for histological evaluation. The tissue sections were deparaffinized with xylene and rehydrated in a graded alcohol series. Endogenous peroxidases were inactivated by incubation with hydrogen peroxide for 15 min. The sections were stained with H&E in accordance with standard methods. For the detection of YAP and TAZ on histological specimens from patients and volunteers, deparaffinized sections were rehydrated in a graded alcohol series. Endogenous peroxidases were inactivated by incubation with hydrogen peroxide for 10 min at room temperature. Antigen retrieval was performed with heated Target Retrieval Solution (pH 6.0). Sections were incubated overnight at 4 °C in a wet chamber with a primary anti-YAP antibody (diluted 1:200; sc-15407, Santa Cruz Biotechnology, Inc., Dallas, TX, USA), anti-TAZ antibody (diluted 1:200; sc-48805, Santa Cruz Biotechnology), or negative control antibody rabbit IgG isotype control (diluted 1:100; GTX35035, GeneTex, Irvine, CA, USA) Horseradish peroxidase-conjugated secondary antibody was detected using the Real Envision System (Dako, Carpinteria, CA, USA). Staining was performed using substrate chromogen solution for 1 min. Counter staining was performed using Mayer’s hematoxylin (Dako). For the detection of VEGF and CD31 on histological specimens from mice, a polyclonal rabbit anti-VEGF primary antibody (diluted 1:1000; Proteintech, Chicago, IL, USA) and a polyclonal rabbit anti-CD31 primary antibody (diluted 1:1000; Abcam, Cambridge, UK) were diluted in antibody diluent (Dako) containing background-reducing components. The sections were incubated with primary antibodies at room temperature for 1 h and proteins were detected using an ImmPress Horseradish Peroxidase Universal Antibody Polymer Detection Kit (Vector Laboratories, Burlingame, CA, USA) for 30 min at room temperature. Staining was performed with diaminobenzidine (Vector Laboratories) for 1 min, and hematoxylin counterstaining was used.

### 4.4. Digital Analysis of Immunohistochemistry Images

Stained tissue samples were digitized using a DMI 5000B light microscope (Leica Microsystems, Wetzlar, Germany). The degrees of expression of YAP and TAZ were based on the semi-quantitative protein expression measurement method [23] and assessed using the free software ImageJ Fiji (version 1.2; WS Rasband, National Institute of Health, Bethesda, MD, http://imagej.net/Fiji/Downloads). The immunostaining densities of stained tissues were determined with primary antibodies against YAP or TAZ, and a rabbit IgG isotype control was used as a negative control. Each value of YAP and TAZ was divided by the rabbit IgG isotype control value and then averaged.

### 4.5. qPCR

Total RNA from tissues was isolated with TRIzol reagent (Invitrogen, Carlsbad, CA, USA) and reverse transcribed to cDNA by using a PrimeScript RT Master Mix Kit (Takara Bio Inc., Otsu, Japan). cDNA was generated using a Thermal Cycler Dice Real Time System TP800 (Takara Bio Inc.) with qPCR SYBR Green to determine the transcriptional expression of specific genes. The qPCR condition was amplified as follows: 50 cycles of denaturation at 9 °C 5 s, annealing at 6 °C 30 s. Glyceraldehyde-3-phosphate dehydrogenase was used for normalization. Relative gene expression was calculated by the 2^ddCt^ method. The primer sequences were as follows: glyceraldehyde-3-phosphate dehydrogenase, (F): 5′-AACAGCAACTCCCACTCTTC-3′ and (R): 5′-CCTGTTGCTGTAGCCGTATT-3′; and VEGF, (F): 5′-AGGCTGCTGTAACGATGAAG-3′ and (R): 5′-TCTCCTATGTGCTGGCTTTG-3′.

### 4.6. Statistical Analysis

Data were analyzed by a one-way analysis of variance, followed by Dunnett’s *t*-test. GraphPad Prism 5 software (GraphPad Software, Inc., La Jolla, CA, USA) was used for most analyses of statistical significance. Paired *t*-tests and a repeated measures analysis of variance to compare the mRNA expression levels of VEGF among groups were performed with SPSS Statistics (SPSS Inc., Chicago, IL, USA). A value of *p* < 0.05 was considered statistically significant.

## Data Availability

The data presented in this study are available on request from the corresponding author. The data are not publicly available due to privacy.

## Abbreviations

YAP/TAZ	Yes-associated protein/transcription activator with a PDZ-binding motif
VEGF	Vascular endothelial growth factor
AMP	Antimicrobial peptides
VP	Verteporfin
